# Wnt signaling related transcripts and their relationship to energy metabolism in C2C12 myoblasts under temperature stress

**DOI:** 10.7717/peerj.11625

**Published:** 2021-06-14

**Authors:** Marua Abu Risha, Asghar Ali, Puntita Siengdee, Nares Trakooljul, Fiete Haack, Dirk Dannenberger, Klaus Wimmers, Siriluck Ponsuksili

**Affiliations:** 1 Institute of Genome Biology, Functional Genome Analysis Research Unit, Leibniz Institute for Farm Animal Biology (FBN), Dummerstorf, Germany; 2 Institute of Muscle Biology and Growth, Leibniz Institute for Farm Animal Biology (FBN), Dummerstorf, Germany; 3 Institute of Genome Biology, Genomics Research Unit, Leibniz Institute for Farm Animal Biology, Dummerstorf, Germany; 4 Faculty of Agriculture and Environmental Science, University Rostock, Rostock, Germany

**Keywords:** C2C12, Energy metabolism, Glycolysis, Oxidative phosphorylation, Heat/cold stress, Wnt signalling, Transcripts

## Abstract

Temperature stress is one of the main environmental stressors affecting the welfare, health and productivity of livestock. Temperature changes can modify cell membrane components, disrupting the crosstalk between the cell and its surroundings by affecting signaling pathways including Wnt signaling pathway, which subsequently disrupts cell energy metabolism. The present study aims to understand the effect of temperature stress on the expression of genes involved in Wnt signaling pathways, and their interaction with energy metabolism in C2C12 myoblasts cells. The C2C12 cells were exposed to cold stress (35 °C), mild heat stress (39 °C) and severe heat stress (41 °C), whereas 37 °C was used as control temperature. Transcript levels of important genes involved in Wnt signaling including *Axin2, Tnks2, Sfrp1, Dkk1, Dact1, Cby1, Wnt5a, Wnt7a, Wnt11, Porcn, Ror2, Daam1*, and *Ppp3ca* were significantly altered under severe heat stress (41 °C), whereas eight Wnt signaling-related transcripts (*Daam1, Ppp3ca, Fzd7, Wnt5a, Porcn, Tnks2, Lrp6,* and *Aes*) were significantly altered under cold stress (35 °C) compared to control. Under heat stress transcripts of the Wnt/β-catenin inhibitors (*Sfrp1, Dkk1*, and *Cby1*) and negative regulators (*Dact1* and *Axin2*) are activated. A positive correlation between oxidative phosphorylation and Wnt-related transcripts was found under high temperatures. Transcripts of the cell membrane receptors, including *Lrp6* and *Fzd7*, and the members of Wnt/Ca^+2^ signaling pathway, including *Ppp3ca* and *Porcn* were downregulated under cold stress. Many Wnt signaling-related transcripts were positively correlated with glycolysis under cold stress. These findings indicate a cross-talk between Wnt signaling and energy metabolism under thermal stress.

## Introduction

Temperature fluctuations are among the environmental stresses that cause many changes in living cells and negatively affect animal production in many aspects. Heat stress negatively impacts meat quality (Zaboli et al., 2018; Imik et al., 2012), animal growth (St-Pierre, Cobanov & Schnitkey, 2003), skeletal muscle hypertrophy (Frier & Locke, 2007). It also changes the cellular metabolism (Zhao et al., 2018) and interferes with cellular signaling (Ganesan et al., 2018; Ganesan et al., 2017). On the contrary, mild heat stress can positively impact the healing of injured muscles (Choi et al., 2016). A previous study showed transcriptional changes under thermal stress (Reed et al., 2017). They showed that cold temperature challenge to satellite cells alters the expression of genes involved in cell signalling/signal transduction, whereas high temperature changes the expression of genes related to muscle system development and cell differentiation (Reed et al., 2017).

Temperature fluctuations trigger a response mechanism in the cells, which is believed to start from the membrane (Török et al., 2014). Temperature changes can lead to modifications of membrane components (Ayuyan & Cohen, 2008), particularly in the regions that play an important role in cell signaling (Mollinedo & Gajate, 2015). Wnt signaling is one of these important pathways. The activation of Wnt signaling requires special receptors on the cell membrane, and Wnt ligands (Driehuis & Clevers, 2017).The main element to transmit canonical Wnt signaling is related to LRP6 phosphorylation (Ozhan et al., 2013), and previous studies confirmed that the phosphorylation occurs in the microdomains (Sakane, Yamamoto & Kikuchi, 2010; Sezgin et al., 2017). Evidence pinpoints mediation of Wnt/ß-catenin signal pathway in hyperthermic stress induced cell injuries of human dermal fibroblast (Jiang, Xue & Wang, 2018).

The Wnt signaling pathway is involved in many biological processes during embryonic development. Several Wnt ligands and key components of Wnt signaling in both canonical and non-canonical pathways are also involved in myogenesis (Cisternas et al., 2014). The canonical pathway depends on β-catenin with a regulatory role in gene transcription, whereas the non-canonical pathway is independent of β-catenin (Komiya & Habas, 2008). The non-conical pathway can either be the planar cell polarity Wnt/PCP pathway which is involved in actin remodeling, cell shape and cytoskeleton regulation, or the Wnt/calcium pathway which regulates calcium level in the cells (Komiya & Habas, 2008). Most of the heat-induced genes affecting the WNT pathway typically inhibit WNT signaling was reported (Sun et al., 2015).

Our previous study has shown significant shifts in the energy metabolism of C2C12 myoblasts under heat and cold stress conditions (Sajjanar et al., 2019). Links between thermal stress response especially affecting signaling pathways and cellular energy metabolism is of our interest. Therefore, the aim of our study was to investigate the transcriptional response of Wnt signaling pathway and its interaction with energy metabolism of C2C12 myoblasts under different temperature stress (35 °C, 39 °C and 41 °C compare to 37 °C). The results indicate a possible crosslink between Wnt signaling and energy metabolism under cellular stress response in skeletal muscle cells.

## Materials & methods

### Cell culture

The mouse skeletal muscle myoblasts cell line C2C12 (ATCC^®^ CRL1772™) was cultivated in 75 cm^2^ flask with Dulbecco’s Modified Eagle’s medium high glucose (DMEM, Gibco) containing 10% fetal bovine serum (FBS, Biochrom) and 1% penicillin-streptomycin at 37 °C under 5% CO_2_ till they reached 80% confluence. The passage 9 cells were detached using 0.125% Trypsin-EDTA (Biochrom) and used for the subsequent experiments. For total RNA, 1 × 10^5^ C2C12 cells per well were seeded and cultured in 6 well-plates. For total membranes isolation 5 × 10^5^ C2C12 cells per 75 cm^2^ flask were seeded and cultured. Other than different temperatures, all cells were incubated at similar conditions. Three independent replicates were performed for each temperature.

### Heat/cold stress treatment

Temperature range where mammals can physiological adapt and survive were set between 35 to 41 °C (cold stress 35 °C, control 37 °C, mild heat stress 39 °C, severe heat stress 41 °C). We used the methods as in our previous study (Sajjanar et al., 2019). Briefly, C2C12 cells were seeded and left to attach on the plastic surface for half hour then cultured at four different temperatures (35, 37, 39 and 41 °C) for 72 h in 6-well plates for genomic analysis and 75 cm^2^ flasks for total membranes protein isolation.

### Bioenergetics assay

As described in our previous study (Sajjanar et al., 2019), two different assays were performed to monitor the changes in mitochondrial and glycolytic functions under temperature stress. The results of these two bioenergetics assays under temperature stress have already been reported (Sajjanar et al., 2019). The bioenergetics data were obtained from the same batch and in the same state of the cells as in the RNA experiments. In this study, we assessed the interaction between these two bioenergetics assays with genes related to Wnt signaling pathway. Briefly, cell energy metabolism under thermal stress conditions was examined by measuring mitochondrial function/oxygen consumption rate (OCR) with the Seahorse Cell Mito Stress Test kit. The Cell Mito Stress Test measures key parameters of mitochondrial function by directly measuring the oxygen consumption rate of cells. This test uses modulators of respiration targeting components of the electron transport chain in the mitochondria to reveal key parameters of metabolic function. The compounds (oligomycin, carbonyl cyanide 4-(trifluoromethoxy) phenylhydrazone (FCCP), and a mix of rotenone and antimycin A) were sequentially added to measure ATP production, maximal respiration, and non-mitochondrial respiration, respectively. The Glycolysis Stress Test is the standard assay for measuring glycolytic function in cells via the direct measurement of the glycolytic activity/extracellular acidification rate (ECAR) with the Seahorse XF Glycolysis kit ECAR in real time. Therefore, glucose, oligomycin, and 2-deoxyglucose were added sequentially to reveal and analyse the key function of glycolytic pathway. Both of these tests revealed how metabolic flux and energy metabolism shift after thermal stress.

### Quantitative real-time expression

Genes were selected based on central components of the Wnt signaling pathway including Wnt ligands, receptors and inhibitors of canonical and non-canonical pathway. Total RNA was isolated from C2C12 cells cultured under different thermal conditions using TRI reagent (Sigma-Aldrich) according to the manufacturer’s instructions, then purified by RNeasy MiniKit (Qiagen). DNaseI treatment was done to remove traces of DNA. Superscript II reverse transcriptase (Invitrogen, Carlsbad, CA, USA) was used to synthesize cDNA. The BioMark HD Real-time PCR System (Fluidigm, South San Francisco, CA, USA) comprising a 48 × 48 dynamic array with an integrated fluidic circuit (IFC) was used for qPCR analyses. The master mix for the samples consisted of 2.25 µl of the STA and Exo-I –treated sample, 2.5 µl of SsoFast EvaGreen supermix with low ROX (Biorad, Hercules, CA, USA), and 0.25 µl DNA binding dye. The master mix for the assay included 2.5 µL assay loading reagent, 2.25 µL DNA suspension buffer, and 0.25 µL of a 100 µM primer solution (forward and reverse) provided in Table 1. The qPCR cycle program was: initial denaturation at 95 °C for 60 s, followed by 30 cycles of 95 °C for 5 s each (denaturation) and 60 °C for 20 s (annealing). The reference genes *Hprt1* and *Mrps27* were used as housekeeping genes that showed no significant changes in any of the heat/cold stress treatments. Data analysis was done by 2 − ΔCt method. All experiments were performed three times independently with three technical replicates each.

**Table 1:** Primers sequences used for amplification in qPCR.

Gene symbol	Ref.NCBI	Fwd Primer 5′->3′	Rev Primer 5′->3′
Hsf1	NM_001331214.1	AACCTGGACAACCTGCAGAC	GGAGGCTCTTGTGGAGACAG
Daam1	NM_001286452.1	GAACACAAGCATGAGCTGGA	AACACCTCCTCAGAGCCAGA
Fzd7	NM_008057.3	ATCATCTTCCTGTCGGGTTG	AAGCACCATGAAGAGGATGG
Ppp3ca	NM_001293622.1	ATATCGACGCACCAGTCACA	AAGGCCCACAAATACAGCAC
Ror2	NM_013846.3	CTTCATTGGGAACCGGACTA	AAGACGAAGTGGCAGAAGGA
Dkk1	NM_010051.3	AATATGCATGCCCTCTGACC	ACGGAGCCTTCTTGTCCTTT
Wnt7a	NM_001363757.1	GCCCACCTTTCTGAAGATCA	CTGAGGGGCTGTCTTATTGC
Wnt11	NM_001285792.1	CAGGATCCCAAGCCAATAAA	GACAGGTAGCGGGTCTTGAG
Wnt5a	NM_001256224.2	AAGCAGGCCGTAGGACAGTA	GCCGCGCTATCATACTTCTC
Porcn	NM_001308474.1	GTCCCTGGCATTCATCACTT	GTGTCGTCCACATCGACATC
Dvl2	NM_007888.4	CAAAGTAACGAGCGTGGTGA	CAGCACTCGTACAGCATCGT
Axin2	NM_015732.4	AACCTATGCCCGTTTCCTCT	CTGGTCACCCAACAAGGAGT
Tnks2	NM_001163635.1	TTCAAGTGCAGCAGAAGGTG	GTGGCCCATTTCAACTAGGA
Sfrp1	NM_013834.3	GCTCAACAAGAACTGCCACA	CTCGGGGAACTTGTCACATT
Lrp6	NM_008514.4	ACAGAGCCCTGACATCATCC	TGATTTGCGACTGAGTTTGC
Csnk1g2	XM_006513011.1	GGAGTACGTGCACACCAAGA	GCGATATGGGATGTGCTTCT
Aes	NM_001276288.1	CGCATCAAAGATGAGTTCCA	CTTCACAATTTCCGCCTGTT
Cby1	NM_028634.3	AAGTTTGAAAACGGCCAGTG	CTGAAAGCATGTCCAGCAGA
Dact1	NM_001190466.1	GGAATCCATGAAGGAAAGCA	CCTCGCTTTTGAAGTCCAAG

### Total membranes isolations

5 × 10^5^ C2C12 cells were seeded and cultured in T75 Flask and incubated at different temperatures. Three independent replicates were performed for each temperature. C2C12 cells were washed with growth media and scraped off with the homogenization buffer (100 mM HEPES, 250 mM sucrose, 4 mM EDTA), 1mM Dithiothreitol (DTT)) containing protease inhibitor (Mini Protease Inhibitor Cocktail; Merck, Darmstadt, Germany). Subsequently, the samples were disrupted in a Potter homogenizer (10 strokes) and then the cell lysates were centrifuged (800 ×g, 3 min) at 4 °C. The supernatant was centrifuged (105 400 ×g, 2 h) (Optima XPN-100, Beckman Coulter, Brea, USA) at 4 °C to obtain the total membrane fraction. The pellet (total cell membranes) was dissolved in homogenization buffer and stored at −80 °C until further preparation. Total membrane fraction isolations were performed in three independent experiments and three technical replicate at each temperature.

### Western blot analysis

The Western blot analyses were performed on total membrane fractions of C2C12 samples and concentrations were measured using Pierce™ BCA Protein Assay Kit (Thermo Scientific, USA). Forty five µg proteins were loaded in each well in the gel (TGX Stain-Free™ FastCast™, BioRad), separated by SDS-PAGE, and then transferred to nitrocellulose membrane with 0.2 µm pore size (catalog no. #1704158, Trans-Blot Turbo, BIO-RAD). The membrane was blocked with 5% milk in Tris buffered saline with Tween-20 (TBST) at room temperature for 45 min. The membrane was then washed with TBST, and then incubated with the primary antibodies against LRP6 (C47E12) antibody (1:1000; catalog no. 3395) and phosphorylated LRP6 (Ser1490) Antibody (1:1000; catalog no. 2568T) at 4 °C for 16 h. The membrane was washed with TBST three times then incubated with 5% milk containing secondary antibodies (Goat Anti-Rabbit IgG H&L (HRP),1:2000, catalog no. ab6721, abcam) for 1 h at room temperature. Last step was by washing the membrane with TBST then incubating with SuperSignal™ West Pico PLUS Chemiluminescent Substrate (catalog no. #34577, Thermo Scientific, USA) Reagent for 1 min. The detection was carried using ChemiDoc Imaging Systems (Bio-rad). We used the total protein amount as a reference to normalize our data. Western blot analysis was performed in three independent replicate at each temperature.

### Data analysis

SAS program was used to analyze the normalized data. For the expression data and Western blot, we applied temperature as fixed effect and replicate as random effect in analysis of variance using proc mixed procedure. An adjusting for multiple comparisons across the Type 3 tests for the fixed effect was calculated using the post hoc Tukey-Kramer test. *P* value < 0.05 was considered statistically significant. Graphs were visualized using GraphPad Prism version 9 (GraphPad Software, La Jolla California USA). Pearson correlation between RNA expression levels of Wnt related genes and energy metabolism values “OCR and ECAR” were calculated and *P* value <0.05 was considered statistically significant.

**Figure 1:**
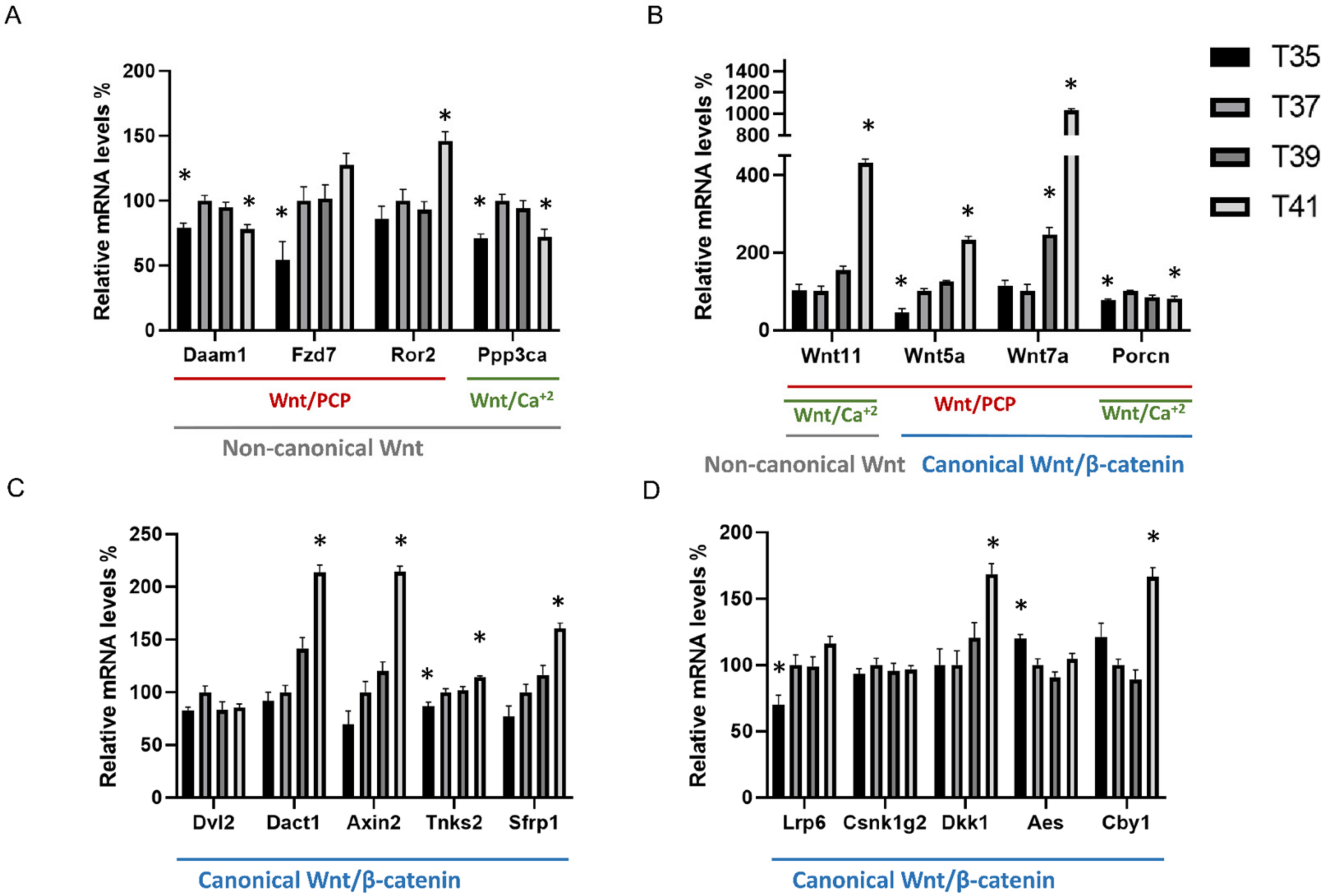
Differentially expressed Wnt-related transcriptions under heat and cold stress. Wnt signaling pathway including non-canonical pathway (gray line) and canonical pathway (blue line) which divides into the planar cell polarity Wnt/PCP pathway (red line) and Wnt/calcium pathway (green line). (A) Wnt related genes belonging to the non-canonical pathway, (B) Wnt related genes belonging to the non-canonical pathway and canonical pathway, (C and D) Wnt related genes belonging to the canonical pathway. Asterisk (*) denotes significant differences between the control group T37 (37 °C) and the group with temperature stress T35, T39 and T41 (35, 39 and 41 °C) respectively at *P* < 0.05. A relative mRNA level of control group was adjusted to 100%. Error bars indicate standard error of the mean (SEM).

## Results

### Transcripts related Wnt signaling under cold/heat stress

Considering the important role of Wnt signaling in cell proliferation, we investigated the effect of cold/heat stress on the expression pattern of Wnt pathway (Fig. 1). The first groups (Fig. 1A) of genes included *Daam1*, *Fzd7*, *Ror2*, *Wnt11*, *Wnt5a*, *Wnt7a* and *Dvl2*, and are known as the components of the non-canonical planar cell polarity pathway (Wnt/PCP). Under thermal stress, the transcript abundance of *Fzd7* was significantly decreased in cold stress whereas *Ror2* transcripts were significantly increased in severe heat stress. *Daam1* was significantly decreased in both cold stress and severe heat stress. Of all Wnt ligand genes, only Wnt5a was significantly decreased in cold stress (35 °C). All Wnt ligands including *Wnt5a*, *Wnt7a* and *Wnt11* were significantly increased in severe heat stress (41 °C) (Fig. 1B). The transcript abundance of *Wnt11* was significantly increased by a factor 4 while *Wnt7a* was significantly increased by a factor of 10. Interestingly, the transcript abundance of *Wnt7a* is very low. Especially at cold or control temperature *Wnt7a* was almost not expressed (ct average = 35) compared to severe heat stress (41 °C) (ct average = 31). No significant change in *Dvl2* expression level between temperatures was detected. Besides *Ppp3ca*, *Wnt5a* and *Wnt11* are also involved in the non-canonical Wnt/Ca+2 pathway. Of these, *Ppp3ca* was significantly decreased at both cold stress (35 °C) and severe heat stress (41 °C) compared to control. The transcripts involved in the Wnt/β catenin signaling pathway are *Wnt5a*, *Wnt7a*, *Dvl2*, *Dact1*, *Axin2*, *Tnks2*, *Sfrp1*, *Lrp6*, *Csnk1g2*, *Aes* and *Cby1*. The transcripts of *Axin2*, *Sfrp1*, *Dact1* and *Cby1* were significantly increased in severe heat stress (Figs. 1C, 1D). *Tnks2* was significantly decreased in cold stress and increased in severe heat stress. *Lrp6* was significantly decreased at cold stress (35 °C) while *Aes* was significantly increased. Additionally, we investigated *Porcn* expression levels which is known to be an important regulator of both canonical and non-canonical Wnt signaling pathway due to its role as a multi-pass transmembrane protein. Transcript abundance of *Porcn* was significantly decreased in both cold stress (35 °C) and severe heat stress (41 °C) compared to control. There was no significant change in *Csnk1g2* RNA expression level between different temperatures. No different RNA expressions of all Wnt ligand genes were found between control (37 °C) and mild heat stress (39 °C) in this study. In addition, under mild heat stress (39 °C), none of the Wnt pathway components were differentially expressed except increased *Wnt7a* transcript.

### Expression pattern of Wnt signaling genes and mitochondrial/ glycolysis functions

**Figure 2:**
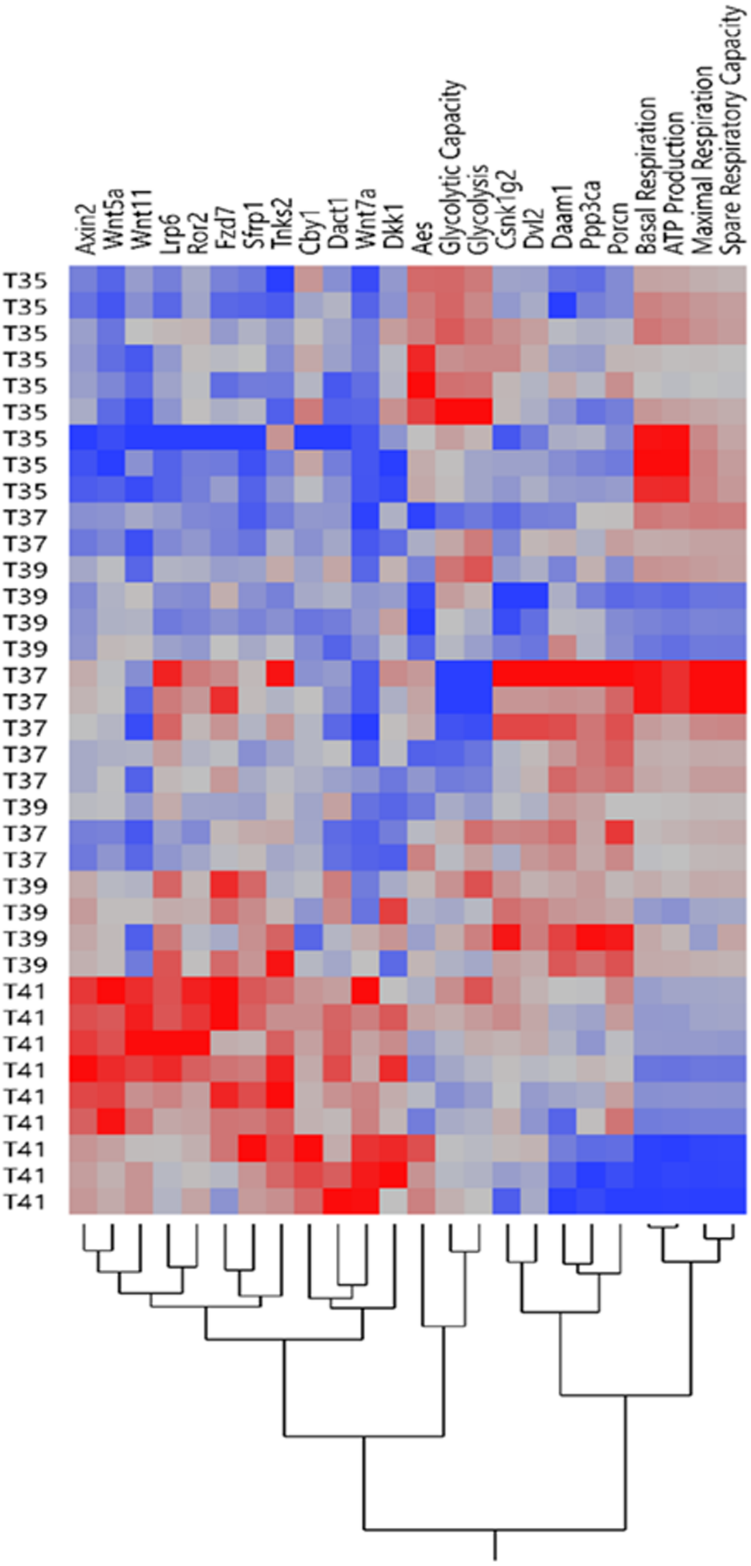
Expression profile of Wnt signaling genes and mitochondrial/glycolysis functions. The heatmap of expression patterns of Wnt signaling genes and mitochondrial/glycolysis functions under temperature stress. Hierarchical clustering of the similar profile of expression and energy metabolism at different temperatures.

Our previous study showed that temperature stress shifted the OXPHOS and the glycolytic functions in muscle cells line (Sajjanar et al., 2019). Expression pattern of Wnt signaling related genes and mitochondrial/glycolysis functions under heat /cold stress is shown in Fig. 2. The heatmap of expression patterns of Wnt signaling genes and mitochondrial/glycolysis functions can be clearly separated under severe heat and cold stress, whereas the pattern was mixed between control and mild temperature (Fig. 2). Under cold stress, glycolysis is the preferential pathway for energy production by promoting glucose metabolism (Sajjanar et al., 2019). Only expression levels of *Aes* was significantly increased under cold stress (Fig. 1D) and cluster together with glycolysis and glycolytic capacity (Fig. 2). Other groups of Wnt related transcripts including *Axin2, Wnt5a, Wnt11, Lrp6, Ror2, Fzd7, Sfrp1, Tnks2, Cby1, Dact1, Wnt7a* and *Dkk1* were clustered together and had lower expression under cold temperature and increased expression during severe heat stress. OXPHOS is significantly downregulated during severe heat stress (Sajjanar et al., 2019), while the expression of transcripts in this group was increased, most of which belong to Wnt inhibitors including *Axin2, Dkk1, Sfrp1, Dact1* and *Cby1.* Transcript levels of *Csnk1g2, Dvl2, Daam1, Ppp3ca,* and *Porcn* were decreased under severe heat and cold stress and these genes belong to both Wnt/PCP and Wnt/Ca^+^ pathways.

### Wnt signaling genes and their relationship to energy metabolism after exposure to Cold/Heat stress

**Figure 3:**
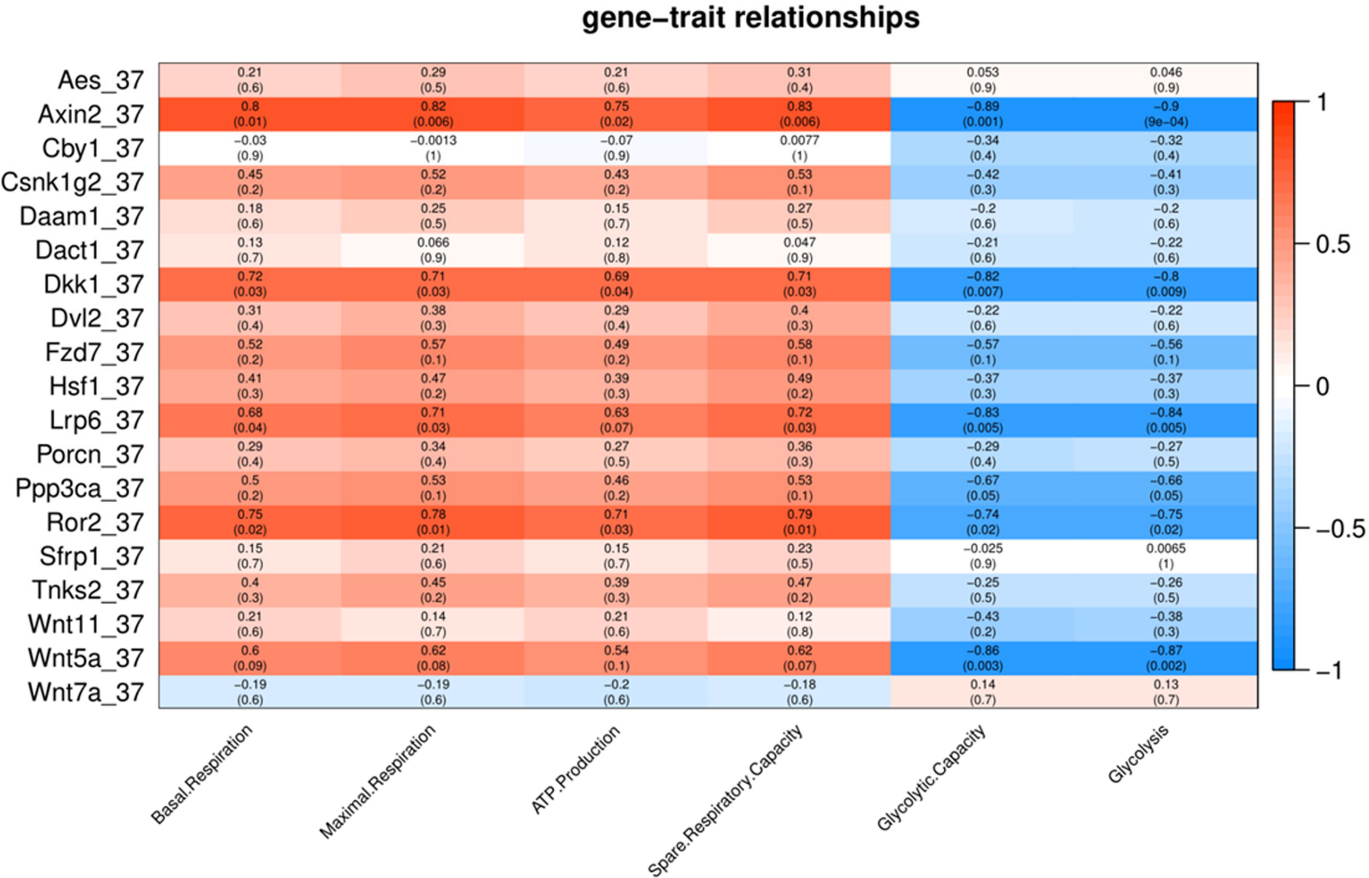
Correlation matrix of Wnt related genes expression and energy metabolism at 37 °C. Number in each cell represents the value of correlation coefficient and the corresponding *p* value. Cell color indicates correlation (red, positive correlation; blue, negative correlation).

To characterize the association of Wnt pathway-related genes with energy metabolism under temperature stress, we applied correlation analyses between gene expression and energy metabolism under various temperature conditions. At control temperature (37 °C) transcript abundance of five genes (*Axin2*, *Lrp6*, *Ror2*, *Dkk1* and *Wnt5a*) showed a negative correlation (*p* < 0.05) with glycolysis and glycolytic capacity (Fig. 3). Four genes (*Axin2*, *Lrp6*, *Dkk1* and *Ror2*) showed a positive correlation with maximal respiration and spare respiratory capacity (Fig. 3). Three genes (*Axin2*, *Dkk1* and *Ror2*) showed a positive correlation with ATP production, while four genes (*Axin2*, *Lrp6*, *Dkk1* and *Ror2*) showed a positive correlation with basal respiration (Fig. 3).

**Figure 4:**
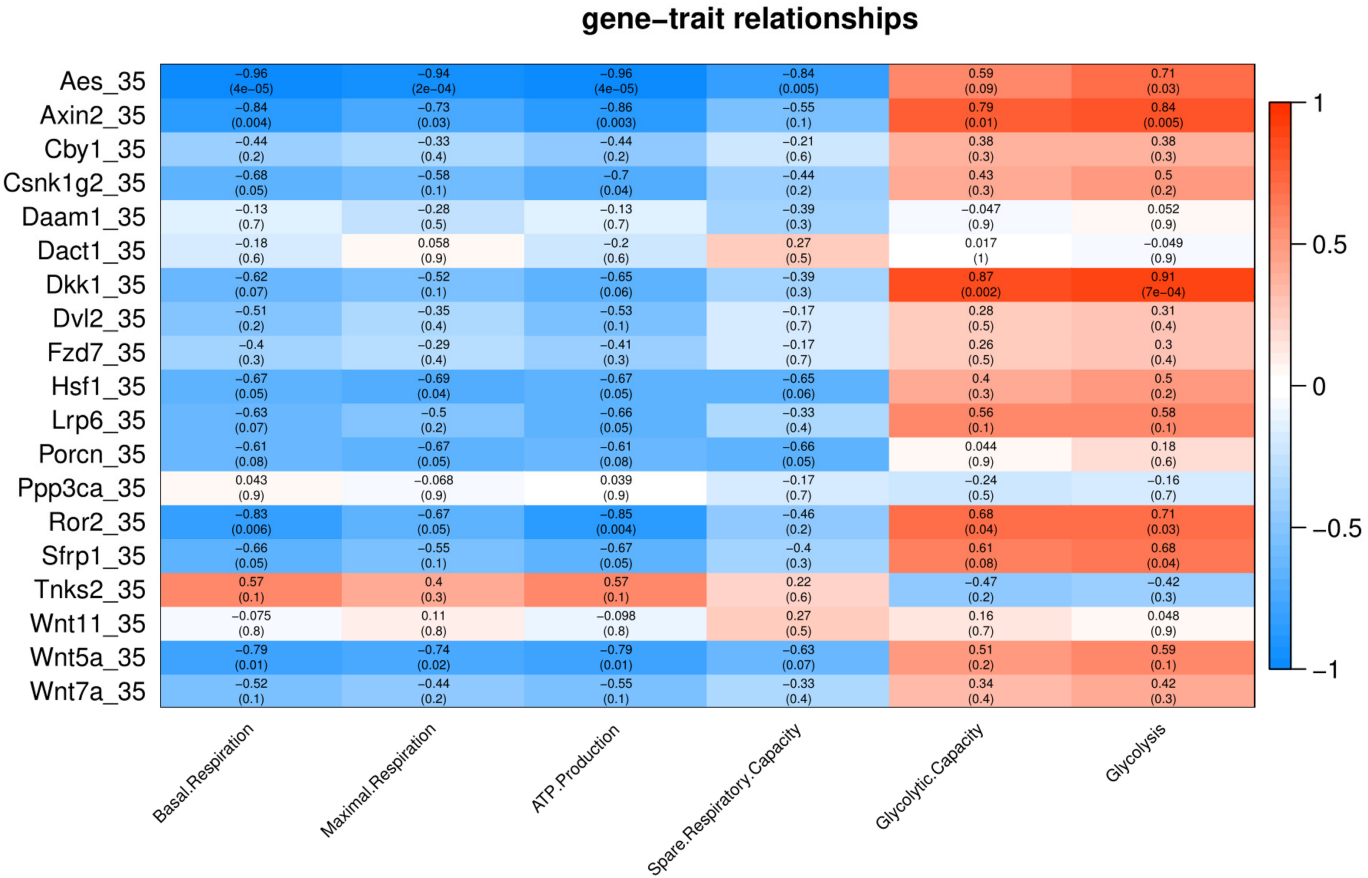
Correlation matrix of Wnt related genes expression and energy metabolism at 35 °C. Number in each cell represents the value of correlation coefficient and the corresponding *p* value. Cell color indicates correlation (red, positive correlation; blue, negative correlation).

Under cold stress (35 °C), five genes (*Aes*, *Axin2*, *Dkk1*, *Ror2*, and *Sfrp1*) showed a positive correlation (*p* < 0.05) with Glycolysis (Fig. 4). Different genes showed a negative correlation with basal respiration (*Aes*, *Axin2*, *Ror2* and *Wnt5a*), maximal respiration (*Aes*, *Axin2*, and *Wnt5a*), ATP production (*Aes*, *Axin2*, *Csnk1g2*, *Ror2* and *Wnt5a*) and spare respiratory capacity (*Aes*) (Fig. 4).

**Figure 5:**
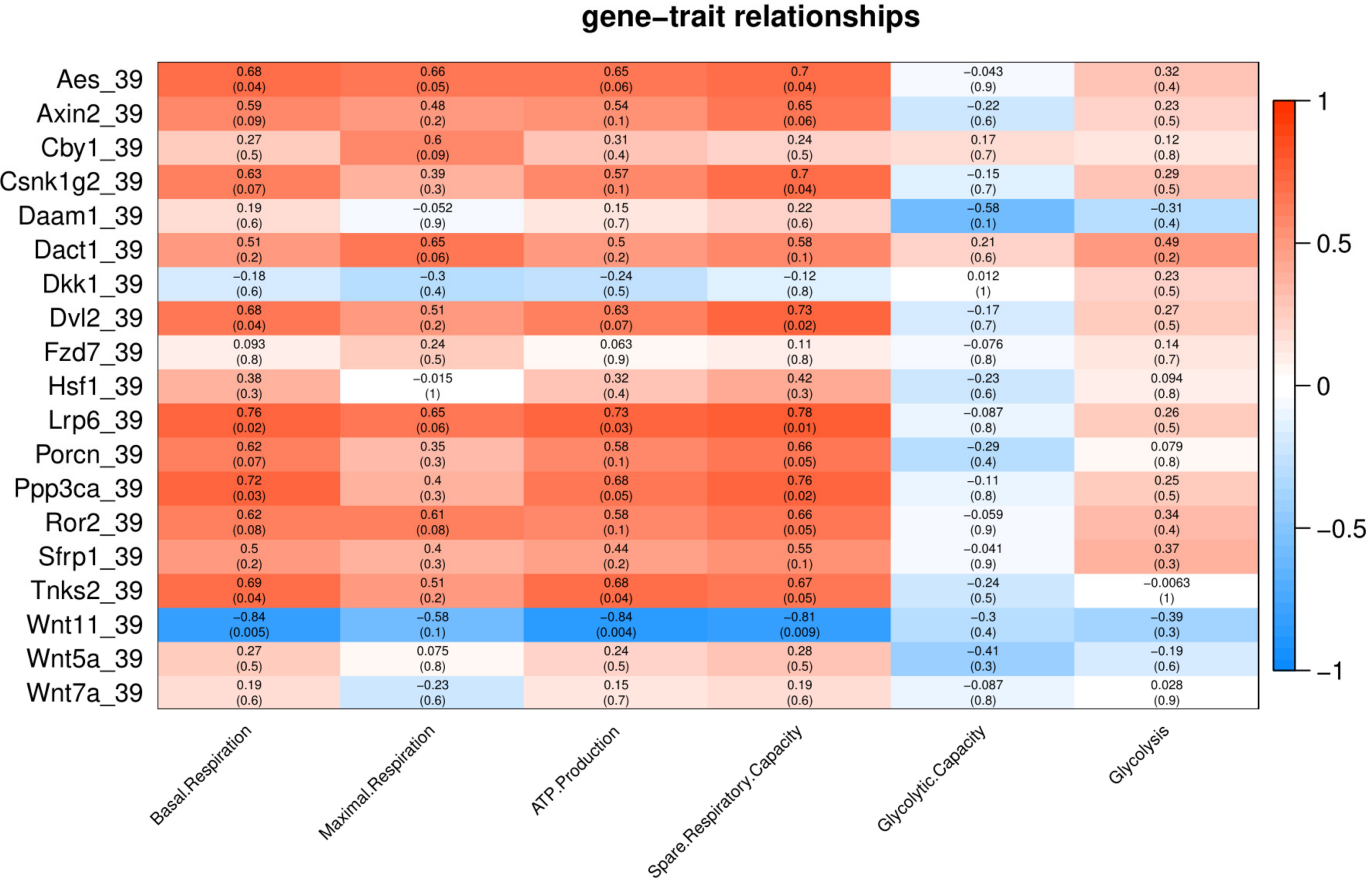
Correlation matrix of Wnt related genes expression and energy metabolism at 39 °C. Number in each cell represents the value of correlation coefficient and the corresponding *p* value. Cell color indicates correlation (red, positive correlation; blue, negative correlation).

Under mild heat stress (39 °C), different genes were positively correlated (*p* < 0.05) with basal respiration (*Aes*, *Dvl2*, *Lrp6*, *Ppp3ca* and *Tnks2*), ATP production (*Lrp6*, and *Tnks2*) and spare respiratory capacity (*Aes*, *Csnk1g2*, *Dvl2*, *Lrp6* and *Ppp3ca*) (Fig. 5). *Wnt11* showed negative correlation (*p* < 0.005) with basal respiration, ATP production, and spare respiratory capacity (Fig. 5).

**Figure 6:**
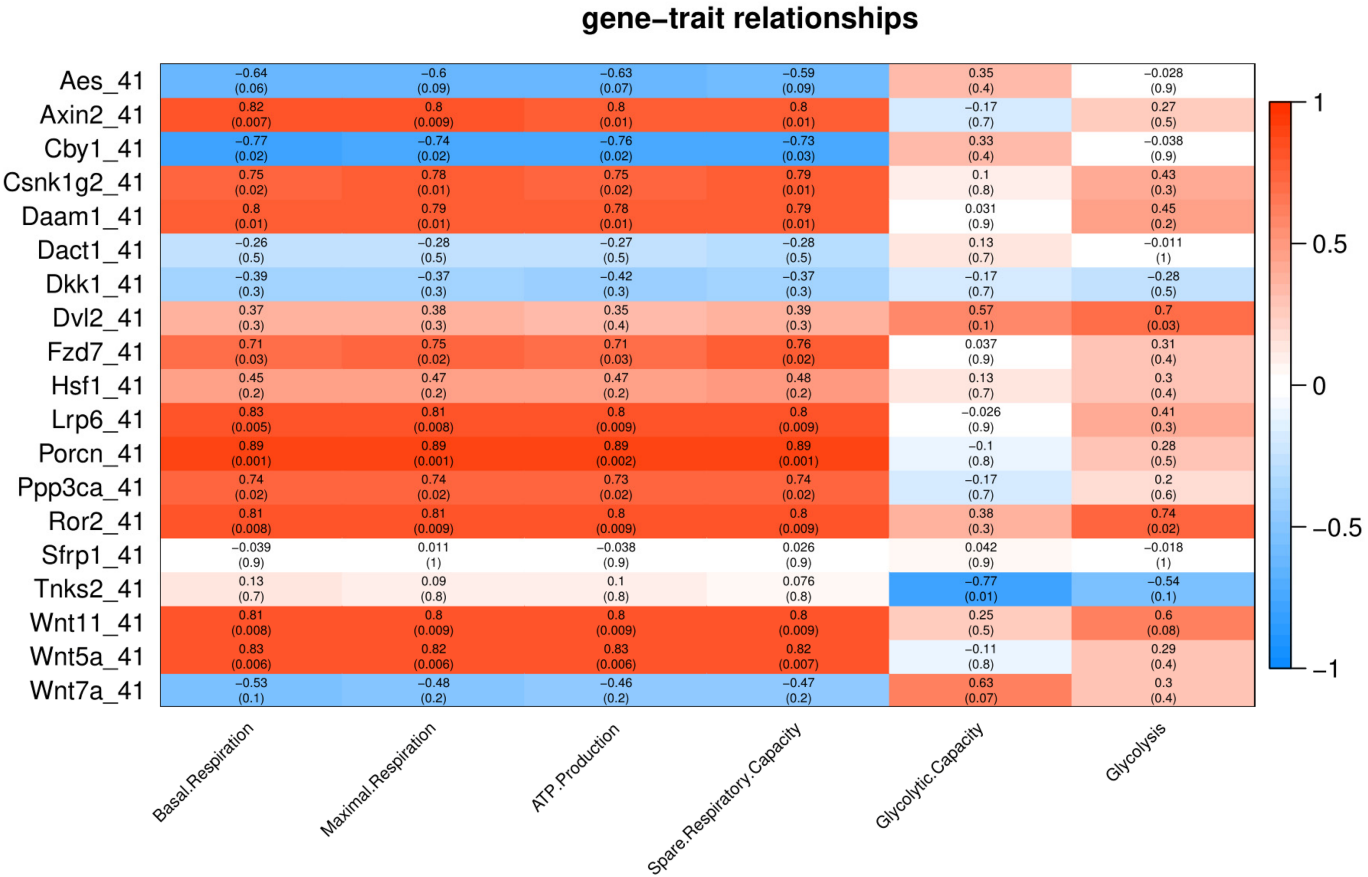
Correlation matrix of Wnt related genes expression and energy metabolism at 41 °C. Number in each cell represents the value of correlation coefficient and the corresponding *p* value. Cell color indicates correlation (red, positive correlation; blue, negative correlation).

Under severe heat stress (41 °C), two genes (*Dvl2* and *Ror2*) showed positive correlation (*p* < 0.05) with Glycolysis, and ten genes (*Axin2*, *Csnk1g2*, *Daam1*, *Fzd7*, *Lrp6*, *Porcn*, *Ppp3ca*, *Ror2*, *Wnt11* and *Wnt5a*) showed positive correlation with basal respiration, maximal respiration, ATP production, and spare respiratory capacity (Fig. 6).Tnks2 showed negative correlation with glycolytic capacity (*p* = 0.01).

### Temperature stress induces phosphorylation of LRP6 on cell membranes

**Figure 7:**
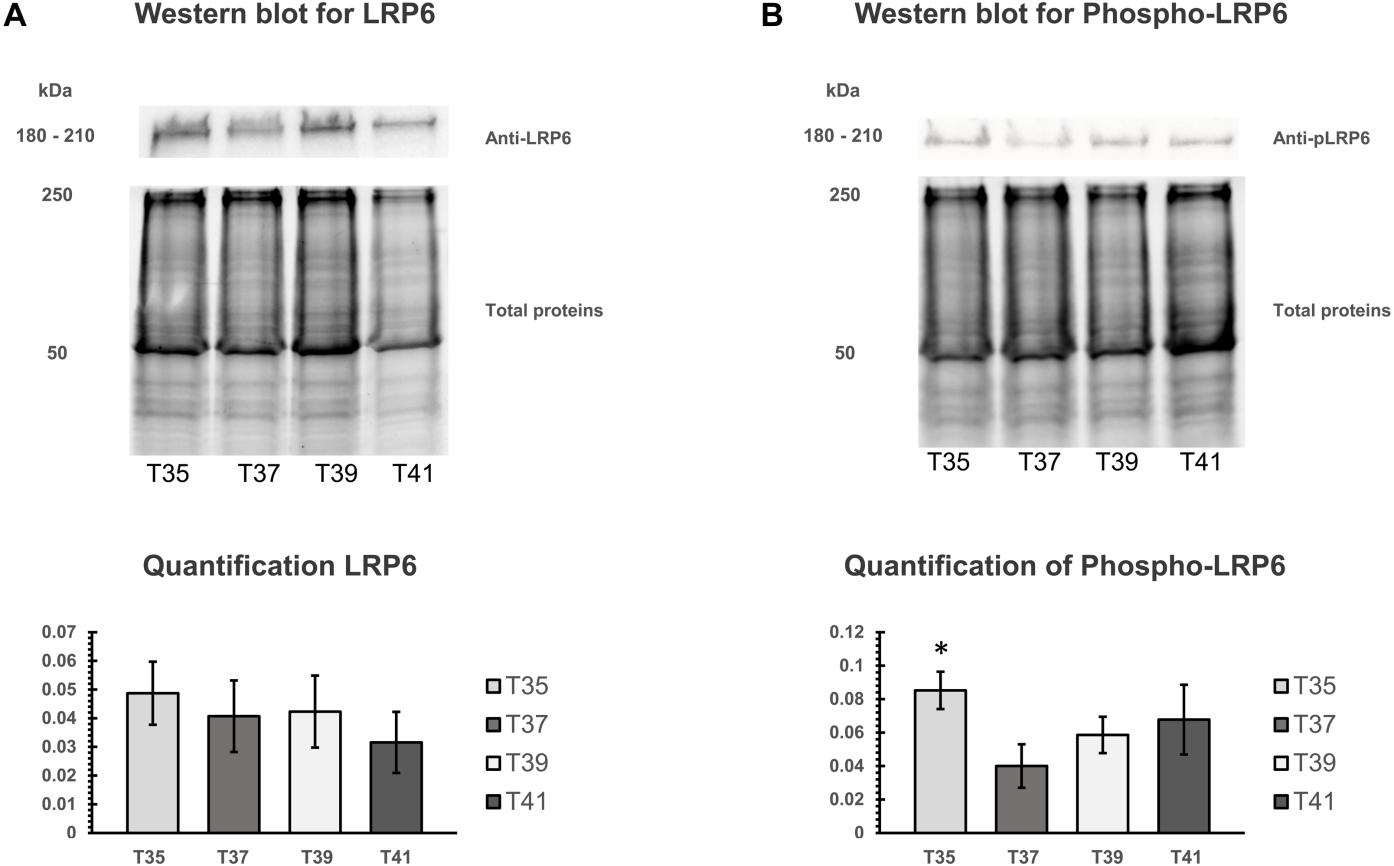
Western blot of LRP6 and phosphorylation of LRP6 under temperature stress. (A) On the upper part, Western blot of total membranes protein of C2C12 using LRP6 antibodies under four different temperatures (T35, T37, T39, and T41) and corresponded of the total membranes proteins, which were used for normalization. The lower part is the quantification of LRP6. (B) On the upper part, Western blot of total membranes protein of C2C12 using phosphorylation LRP6 antibodies under four different temperatures (T35, T37, T39, and T41) and corresponded of the total membranes proteins, which were used for normalization. The lower part is the quantification of phosphor-LRP6. Error bars indicate standard error of the mean (SEM). * indicates *p* < 0.05 compared to the control group 37 °C.

The activation of canonical Wnt signaling pathway requires a specific receptor in cell membranes and Wnt ligands. LRP6 protein is an important receptor in Wnt signaling located on cell membrane. We isolated the cell membranes from cells cultured at different temperatures and measured protein quantity of LRP6 and phospho-LRP6 by Western blot analysis. Total membrane protein was used for normalizing (Figs. 7A, 7B). Three independent experiments were carried out. We found no significant change under heat/cold stress by using LRP6 (Fig. 7A), whereas phosphorylated LRP6 protein was significant higher under cold stress (Fig. 7B). We also found that cells cultured under severe heat stress showed tendency of increased phosphorylated LRP6 protein (Fig. 7B).

## Discussion

Environmental stress including cold/heat stress induces disturbance in the cellular energy metabolism and influences multiple types of signaling molecules including Wnt signaling. The involvement of Wnt signaling during cell proliferation can happen via two pathways, the canonical pathway by mediating the mitosis stages (Kaldis & Pagano, 2009) and the non-canonical pathway which leads to actin polymerization and cytoskeleton regulation (Kim & Davidson, 2011; Dzamba et al., 2009). In this study, selected genes involved in Wnt signaling pathway were evaluated under thermal stress conditions.

### Transcripts related Wnt/PCP, Wnt/Ca2+ and Wnt/β-catenin under cold/heat stress

Wnt/PCP pathway involves several proteins. FZD7 favors non-canonical signaling, and its downregulation restrains cell proliferation (Xue et al., 2018). DAAM1 is required for organizing the cytoskeleton (Habas & He, 2001). Downregulation of *Daam1* or the knockdown of *Wnt5a* inhibits migration and invasion of breast cancer cells (Xiong et al., 2018; Prasad et al., 2016). The regulation of Wnt/PCP pathway can also be done via JNK pathway (Yamanaka et al., 2002). Previous studies indicated that Wnt5a and Ror2 activate the noncanonical Wnt signaling pathway, as determined by activation of JNK in cultured cells (Oishi et al., 2003). Here we found overexpression of *Ror2* and *Wnt5a* transcripts together with the down regulation of *Daam1* transcript under heat stress. At the same time transcript levels of *Wnt11*, *Wnt5a* and *Wnt7a* were highly increased under heat stress, which may cause an increased intracellular accumulation of Wnt ligands in C2C12 cells.

In this study, two key genes in Wnt/Ca2+ pathway (*Ppp3ca* and *Porcn*) were significantly downregulated under both heat/cold stress. *Ppp3ca* belongs to the Wnt/ca2+ pathway, which can be activated through *Wnt11* and *Wnt5a* (Witze et al., 2013). The *Porcn* protein has an important role in Wnt ligand trafficking. It modifies Wnt ligands by adding Palmitoleate which is critical for the Wnt secretion. Depleting *Porcn* will cause intracellular accumulation of Wnt ligands (Barrott et al., 2011).

Wnt/β-catenin is a complicated pathway involving several elements from proteins to transcription factors. During myoblast proliferation, blockade of WNT/β-catenin signaling decreases cell proliferation but does not induce cell death (Suzuki, Pelikan & Iwata, 2015). Real-time determination of cell viability from our previous study using the xCelligence system showed that proliferation of C2C12 was slightly impaired under cold treatment conditions and cell viability was significantly reduced under high thermal stress (Sajjanar et al., 2019). We found lower transcript levels of Lrp6 on the cells under cold stress. Since the LRP6 receptor is located on the cell membrane, we therefore isolated the protein from the cell membrane. No significant increase in LRP6 protein level was found under temperature stress. In contrast, we found that cold stress induces phosphorylation of LRP6 at the cell membrane and possibly triggers further Wnt signaling cascades. Previous studies also confirmed that phosphorylation occurs in microdomains (Sakane, Yamamoto & Kikuchi, 2010; Sezgin et al., 2017). Lypd6 appears to regulate Lrp6 activation, particularly in membrane rafts, which is essential for downstream signaling (Ozhan et al., 2013). The membrane can change its fluidity under the influence of variable temperature (Polezhaev & Volkov, 1979). High temperature tends to increase the distance between membrane phospholipids which will increase the fluidity, while low temperature does the opposite (Fan & Evans, 2015). In our study, a downregulation of *Lrp6* and *Fzd7* transcripts in C2C12 cell under cold stress was found whereas phosphorylated LRP6 protein was upregulated in the cell membrane. The change of these receptors under cold stress may disturb the activation of the Wnt/β-catenin. *Aes* (amino-terminal enhancer of split or Tle5) is co-repressor in Tle family which mediates repression of WNT target genes (Brantjes et al., 2001). It is important to note that the temperature influence on the canonical Wnt signal regulation was negative in both heat and cold, but in two different approaches. Under cold stress, the expression of Wnt ligands was reduced while in heat stress we noticed many Wnt inhibitors being overexpressed along with some negative regulators. AXIN2 is considered a negative regulator of the canonical pathway, as it promotes the degradation of β-catenin (Jho et al., 2002; Wu et al., 2012). DACT1 is another negative regulator of the canonical pathway which can mediate dishevelled degradation (Zhang et al., 2006) and inhibit cell proliferation (Zhu et al., 2017). The role of CBY1 in the canonical pathway covers the deactivation of the β-catenin transactivation complex (Takemaru & Moon, 2003). It has been reported that Cby overexpression decreased β-catenin activity (Singh et al., 2007). SFRP1 is an antagonist of Wnt3 where it binds and prevents the activation of the pathway (Yang et al., 2015) and it can promote apoptosis by suppressing proliferation, migration and invasion (Wang et al., 2018). Previous study reported that the transcripts in Wnt signalling pathways including SFRP1 and DACT1 was increased by heat treatment, similar to what we found in this study(Sun et al., 2015). A recent study in IPEC-J2 cells showed downregulation Wnt/β-catenin pathway at 41 °C, and the protein level of its downstream targets AXIN2, GSK3 β, β-catenin, cyclin D1, and c-Myc were also disrupted (Zhou et al., 2020). Our results clearly demonstrate an overexpression of inhibitors (*Sfrp1*, *Dkk1*, and *Cby1*) and negative regulators (*Dact1* and *Axin2*) of Wnt/β-catenin pathway in the high temperature (41 °C). This may indicated that WNT pathways appears to be promoting cell survival during heat stress by suppressing proliferation.

### Wnt signaling genes and their relationship to energy metabolism after exposure to Cold/Heat stress

Environmental changes like temperature also play a significant role in energy metabolism, as indicated some previous studies (Sajjanar et al., 2019; Little & Seebacher, 2016; Mitov et al., 2017). We have previously reported that in heat stress, reduced maximal respiration and spare respiratory capacity to promote cell survival, whereas in cold stress, cells prefer glycolysis as a rapid compensatory mechanism to meet the energy demand as an adaptive thermogenic response (Sajjanar et al., 2019). In current study, we found that many Wnt related transcripts under cold stress were positively correlated with glycolysis. Aes was positively correlated with glycolysis under cold stress and cluster with glycolysis. A previous study reported that defects in Wnt signalling may determine a metabolic switch in fuel utilization towards glycolysis (Chafey et al., 2009).

Specific correlation patterns between mitochondrial or glycolytic functions and Wnt signaling pathway-related genes were found in this study. Some transcripts, including *Axin2, Ror2 and Wnt5a*, were positively correlated with mitochondrial traits in the control and severe heat stress, but negatively in cold stress. A group of genes, including *Csnk1g2, Daam1, Fzd7, Porcn, Ppp3ca* and *Wnt11*, correlated positively with mitochondrial traits only in severe heat stress. Wnt signalling is known to promote cell proliferation. To promote cell survival under heat stress, Wnt signaling pathway is activated and there is a change in the expression of Wnt/β-catenin inhibitors, Wnt/PCP, Wnt ligands and Wnt antagonists. We also observed the genes involved in Wnt signaling are positively correlated with energy metabolism. These results indicate that activated Wnt inhibitors or the Wnt antagonists together with reduce metabolic flux promote cell survival under heat stress.

The role of Wnt/β-catenin signaling in mitochondrial biogenesis has been previously reported (An et al., 2010). The regulation of Wnt/β-catenin pathway can be carried out by TNKS1 and TNKS2 (Huang et al., 2009). By controlling AXIN1 and AXIN2 levels and suppressing Wnt/β-catenin signaling, both TNKS1/2 regulate glycolysis (Li et al., 2015). *Ror2* overexpression with *Wnt5a* stimulation and *Ror1* knockdown showed that the active non-canonical pathway targets modify other pathways that are involved in cell metabolism like glycolysis and fatty acid metabolism (Bayerlová et al., 2017).

A previous study in Human melanoma cell lines (A2058 and HTB63 showed a positive correlation between *Wnt5a* and lactic dehydrogenase expression, indicating the role of *Wnt5a* in glycolytic flux stimulation in cancer cell metabolism (Sherwood et al., 2014). Together, the correlation between Wnt related genes and energy traits was shifted between temperatures, in particular *Axin2*, *Ror2*, *Lrp6* and *Wnt5a* had an opposite correlation between the cold stress and the severe heat stress. These findings indicate potential cross-talks between Wnt signaling and energy metabolism.

### Conclusion

In summary, we demonstrated a relationship between Wnt related genes and energy metabolism including oxidative phosphorylation and glycolysis under cold/heat stress in C2C12 muscle cell line. Cold stress and severe heat stress lead to transcriptional responses to the regulation of Wnt-related genes in different ways. Thermal stress activates Wnt signaling in C2C12 cells in particular the transcripts of Wnt/β-catenin inhibitors, Wnt/PCP pathway and Wnt ligands are activated under heat stress. Cell membrane receptors *Lrp6* and *Fzd7* are activated under cold stress. A positive correlation between oxidative phosphorylation and Wnt-related transcripts was found under high temperature while negative correlation was found under cold temperature. More experiments are needed to make definitive conclusions about the relationship between these pathways *in vivo* under non-diseased/cancerous states.

##  Supplemental Information

10.7717/peerj.11625/supp-1 Supplemental Information 1 Raw data of the RT-PCR data Each temperature was performed independently for three replicates, each replicate was performed in three wells, and each sample (well) was performed in two technical replicates.

10.7717/peerj.11625/supp-2 Supplemental Information 2 Original Western blot image for total protein and correspondence blot of LPR6 protein from gel 1, gel 2, and gel 3 All Figure was carried using ChemiDoc Imaging Systems (Bio-rad). We used the total protein amount as a reference to normalize LPR6 protein data. Western blot analysis was performed in three independent experiments.

10.7717/peerj.11625/supp-3 Supplemental Information 3 Raw data of Fig. 2 The detection was carried using ChemiDoc Imaging Systems (Bio-rad). We used the total protein amount as a reference to normalize LPR6 protein data. Western blot analysis was performed in three independent experiments.

## Definitions

Daam1: Dishevelled Associated Activator Of Morphogenesis 1
Fzd7: Frizzled Class Receptor 7
Ppp3ca: Protein Phosphatase 3 Catalytic Subunit Alpha
Ror2: Receptor Tyrosine Kinase Like Orphan Receptor 2
Dkk1: Dickkopf WNT Signaling Pathway Inhibitor 1
Wnt7a: Wnt Family Member 7A
Wnt11: Wnt Family Member 11
Wnt5a: Wnt Family Member 5A
Porcn: Porcupine O-Acyltransferase
Dvl2: Dishevelled Segment Polarity Protein 2
Axin2: Axin 2
Tnks2: Tankyrase 2
Sfrp1: Secreted Frizzled Related Protein 1
Lrp6: LDL Receptor Related Protein 6
Csnk1g2: Casein Kinase 1 Gamma 2
Aes: TLE Family Member 5, Transcriptional Modulator
Cby1: Chibby Family Member 1, Beta Catenin Antagonist
Dact1: Dishevelled Binding Antagonist Of Beta Catenin 1
JNK: c-Jun N-terminal kinases

## Additional Information and Declarations

### Data Availability

The following information was supplied regarding data availability: Raw data are available as Supplemental Files.
