# Supplementary figures and images for: Wnt signaling related transcripts and their relationship to energy metabolism in C2C12 myoblasts under temperature stress
Source: PeerJ. 2021 Jun 14;9:e11625. doi: 10.7717/peerj.11625 (PMC8210811; doi:10.7717/peerj.11625)

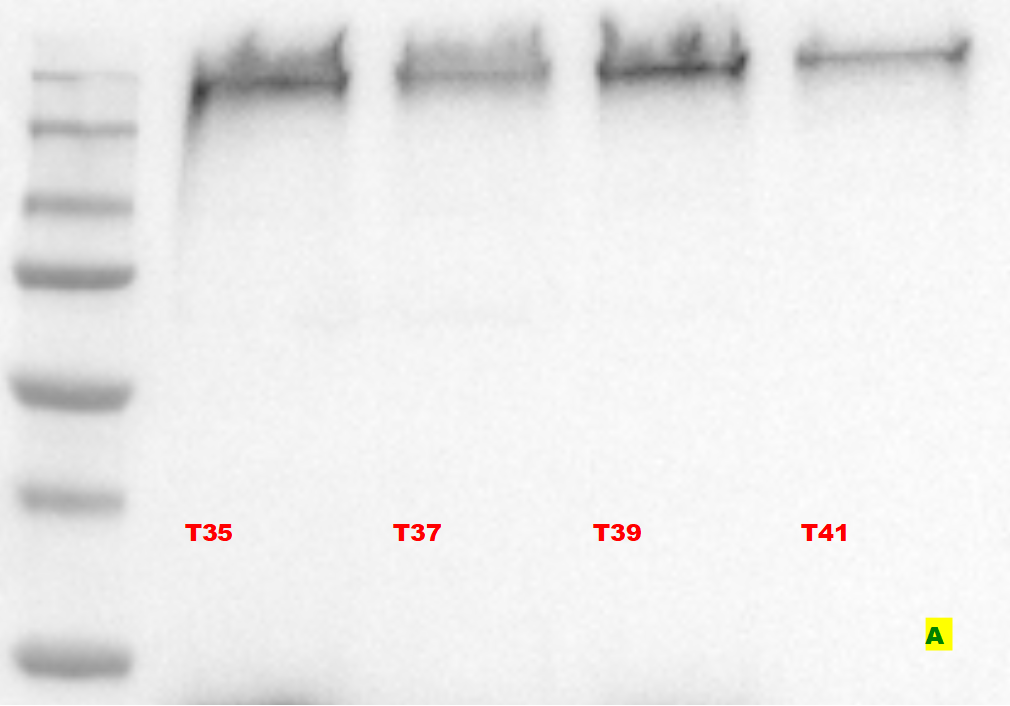

Supplement: Supplemental Information 2 — All Figure was carried using ChemiDoc Imaging Systems (Bio-rad). We used the total protein amount as a reference to normalize LPR6 protein data. Western blot analysis was performed in three independent experiments. [file peerj-09-11625-s002.zip › M1_Anti_LRP6.tif]

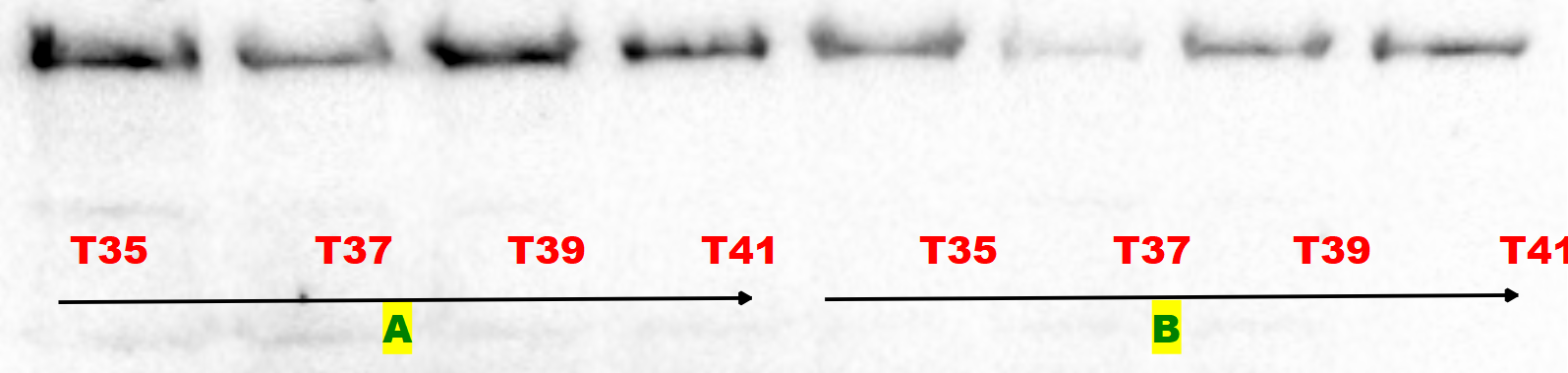

Supplement: Supplemental Information 2 — All Figure was carried using ChemiDoc Imaging Systems (Bio-rad). We used the total protein amount as a reference to normalize LPR6 protein data. Western blot analysis was performed in three independent experiments. [file peerj-09-11625-s002.zip › M1_Anti_Phospho LRP6.tif]

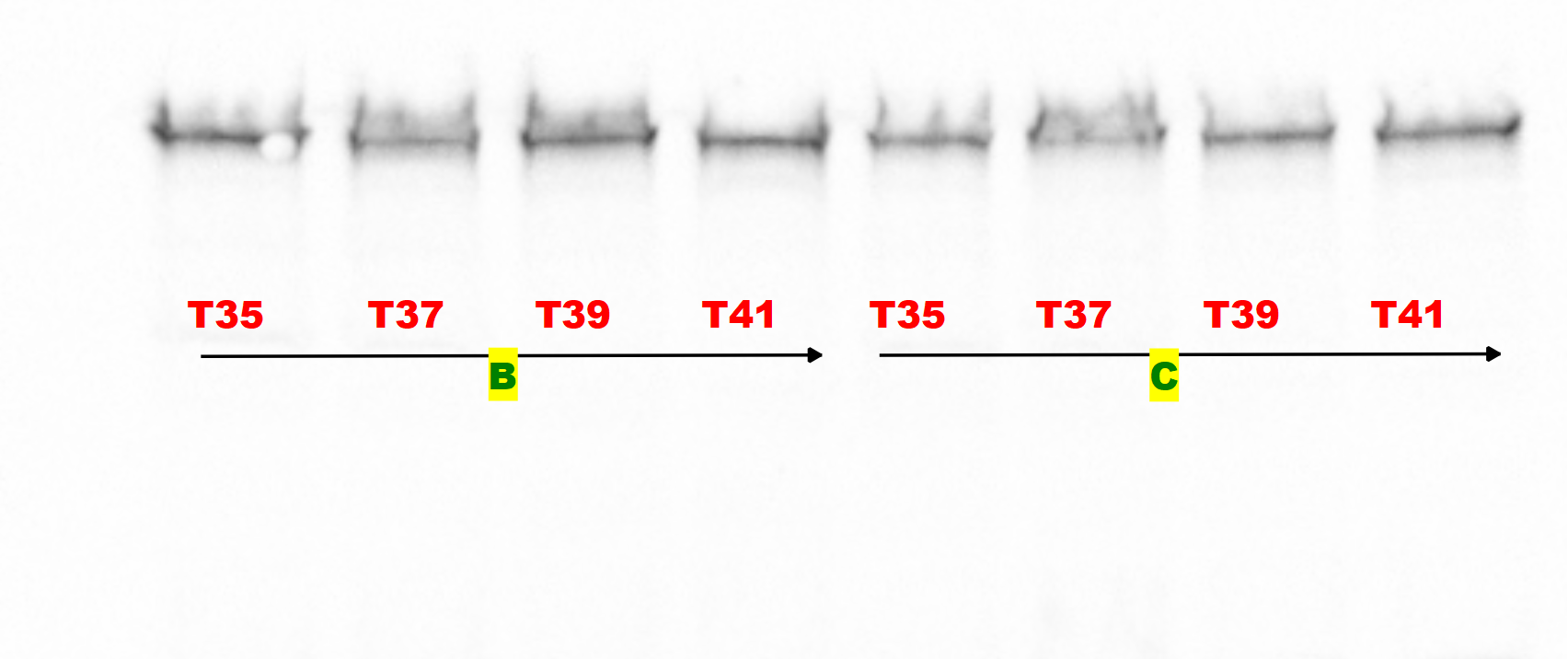

Supplement: Supplemental Information 2 — All Figure was carried using ChemiDoc Imaging Systems (Bio-rad). We used the total protein amount as a reference to normalize LPR6 protein data. Western blot analysis was performed in three independent experiments. [file peerj-09-11625-s002.zip › M2_Anti_LRP6.tif]

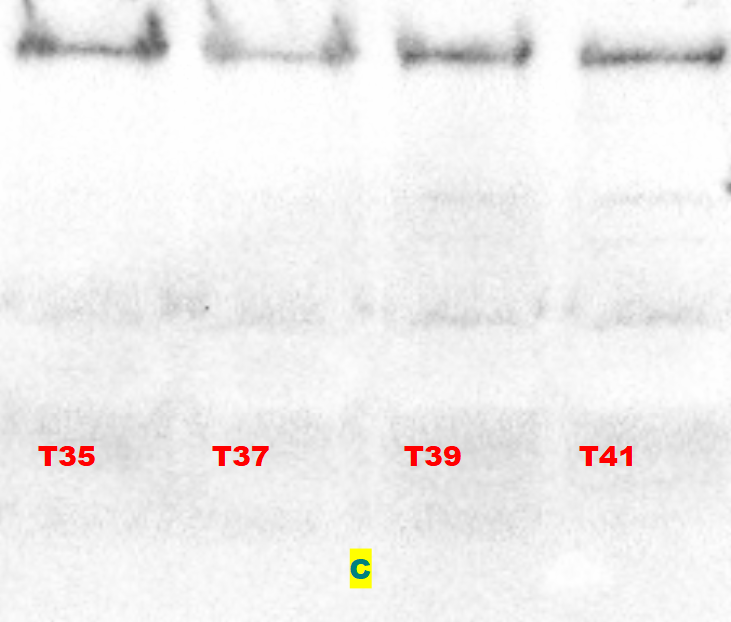

Supplement: Supplemental Information 2 — All Figure was carried using ChemiDoc Imaging Systems (Bio-rad). We used the total protein amount as a reference to normalize LPR6 protein data. Western blot analysis was performed in three independent experiments. [file peerj-09-11625-s002.zip › M2_Anti_Phospho LRP6.tif]

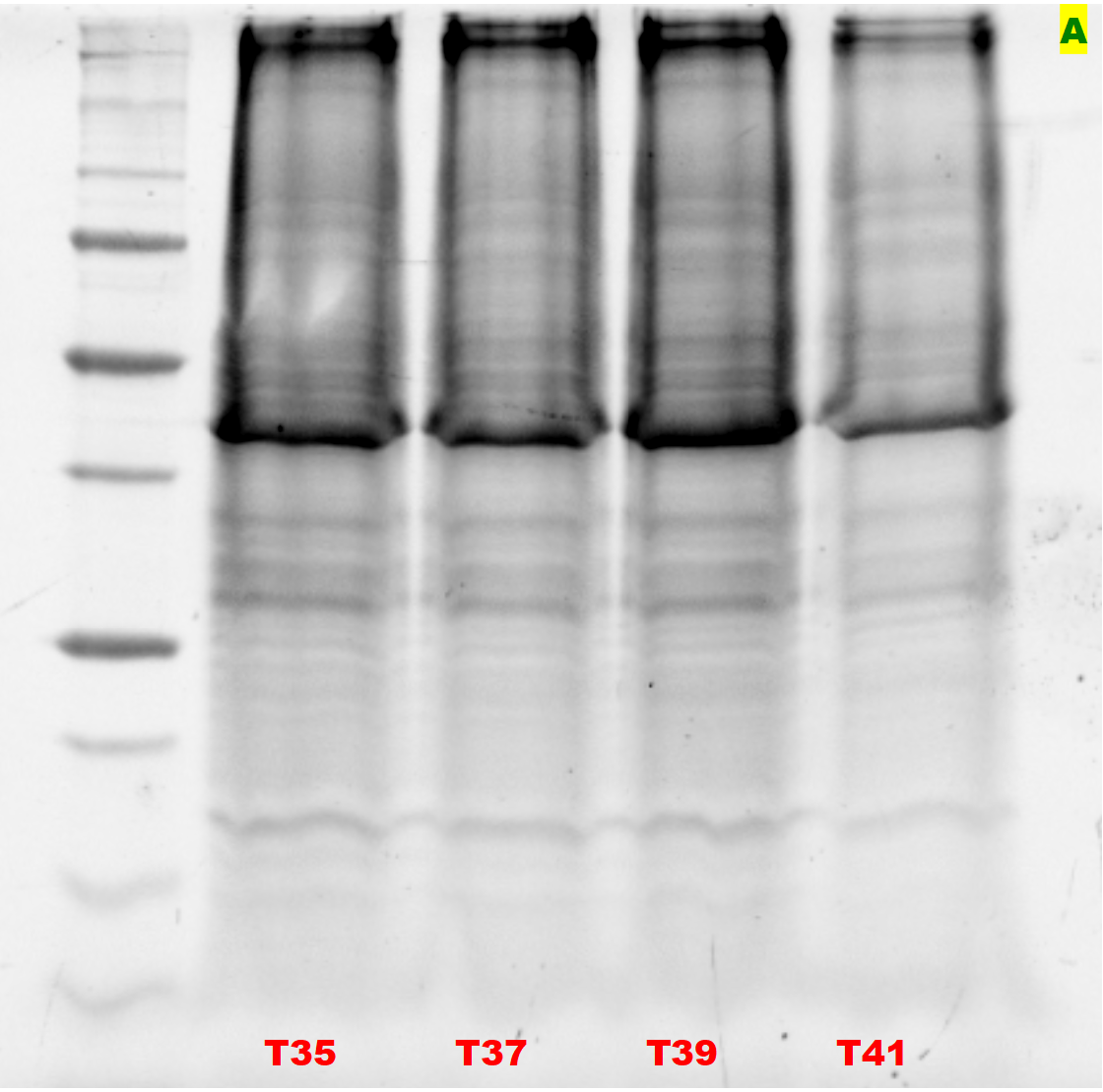

Supplement: Supplemental Information 2 — All Figure was carried using ChemiDoc Imaging Systems (Bio-rad). We used the total protein amount as a reference to normalize LPR6 protein data. Western blot analysis was performed in three independent experiments. [file peerj-09-11625-s002.zip › Gel1_LRP6.tif]

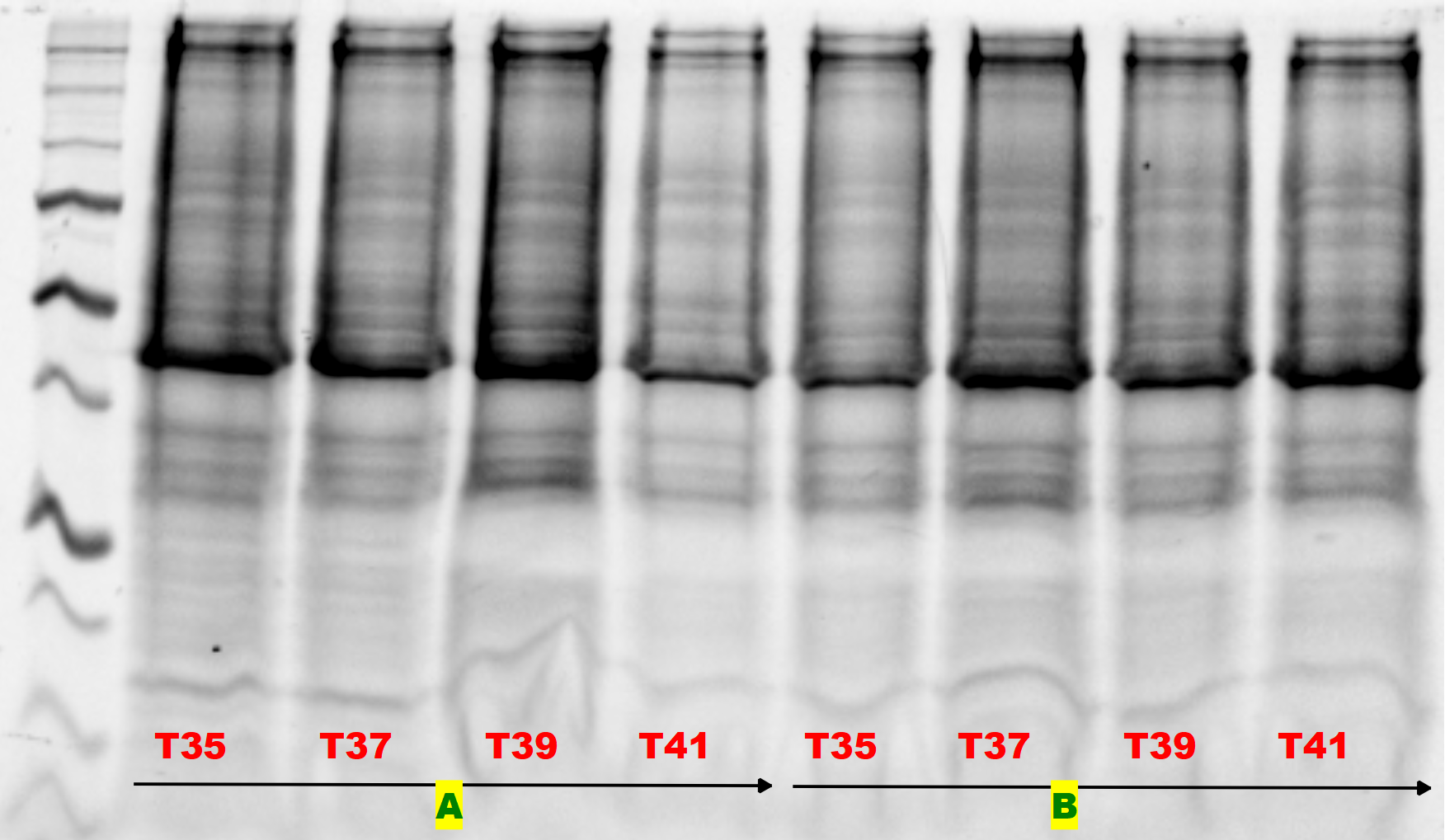

Supplement: Supplemental Information 2 — All Figure was carried using ChemiDoc Imaging Systems (Bio-rad). We used the total protein amount as a reference to normalize LPR6 protein data. Western blot analysis was performed in three independent experiments. [file peerj-09-11625-s002.zip › Gel1_Phospho LRP6.tif]

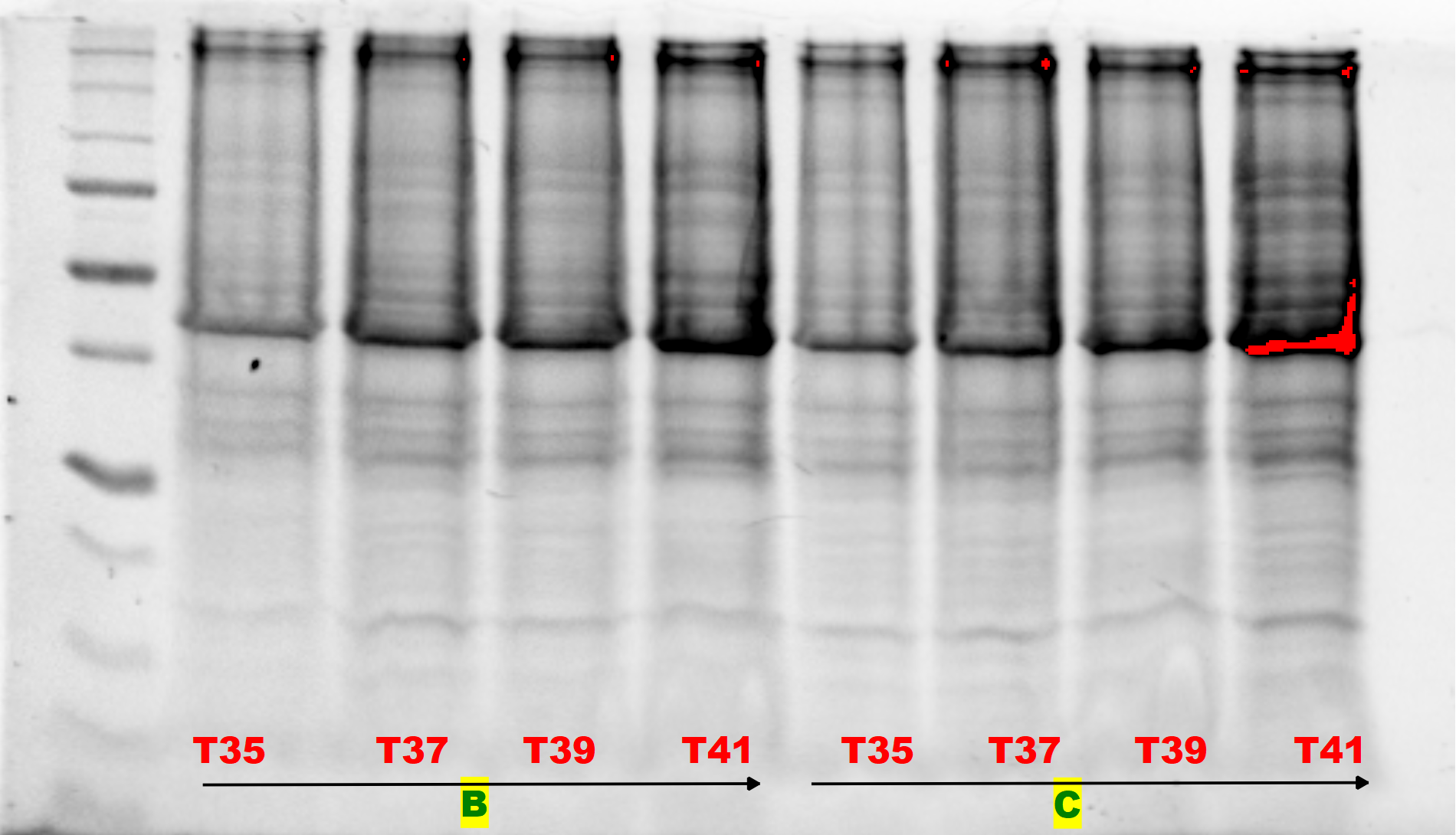

Supplement: Supplemental Information 2 — All Figure was carried using ChemiDoc Imaging Systems (Bio-rad). We used the total protein amount as a reference to normalize LPR6 protein data. Western blot analysis was performed in three independent experiments. [file peerj-09-11625-s002.zip › Gel2_LRP6.tif]

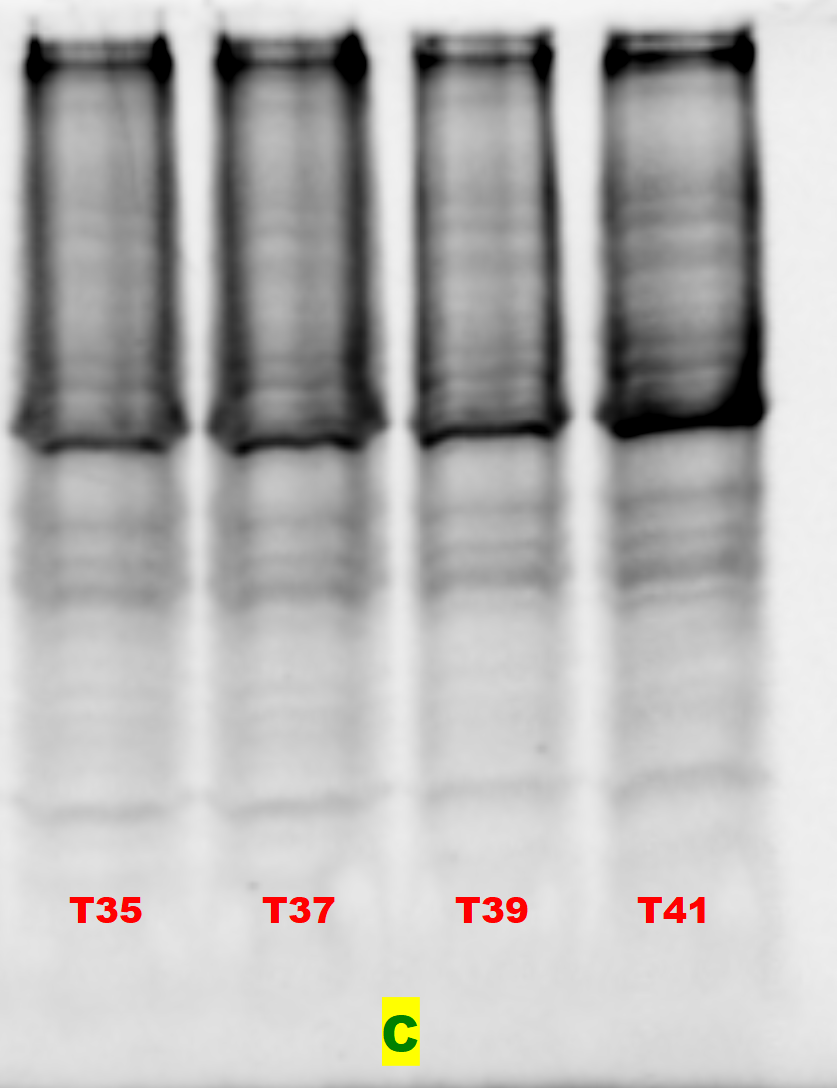

Supplement: Supplemental Information 2 — All Figure was carried using ChemiDoc Imaging Systems (Bio-rad). We used the total protein amount as a reference to normalize LPR6 protein data. Western blot analysis was performed in three independent experiments. [file peerj-09-11625-s002.zip › Gel2_Phospho LRP6.tif]
